# High-Efficiency Prediction of Surfactant Properties through Automated Hydrophilic–Hydrophobic Partitioning

**DOI:** 10.1021/acs.jcim.6c00398

**Published:** 2026-06-02

**Authors:** Sofía González-Núñez, Carlos Amador, Mariano Martín

**Affiliations:** † Departamento de Ingeniería Química. 16779 Universidad de Salamanca. Pza. Caídos 1-5, Salamanca 37008, Spain; ‡ Newcastle Innovative Centre. 201091 Procter and Gamble. Whitley Rd, Longbenton, Newcastle Upon Tyne Tyne And Wear NE12 9SR, England

## Abstract

Predicting surfactant properties from structurally diverse molecules remains challenging and limits the rational design of new surfactants. This difficulty arises from the complex interplay between hydrophilic head and hydrophobic tail domains, which govern self-assembly and interfacial behavior. This work presents an automated decomposition-based framework that identifies surfactant head and tail domains and computes interpretable, domain-specific structural descriptors. These descriptors are used in data-driven models to predict key properties, namely critical micelle concentration (CMC), surface tension, and adsorption efficiency, across a wide range of surfactants, including Gemini architectures. Accurate predictions with reduced computational cost and improved extrapolation are achieved. The same descriptors enable clustering analysis of head and tail structures to identify combinations that yield optimal property profiles, supporting the rational design of new surfactants with targeted functionalities.

## Graphical abstract

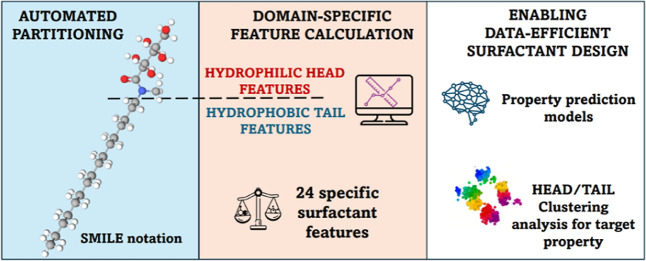

## Introduction

Surfactants, short for surface-active agents, are compounds capable of reducing the interfacial tension between two phases. They are amphiphilic molecules characterized by the presence of distinct hydrophilic (polar) and hydrophobic (nonpolar) moieties, which govern their self-assembly, interfacial behavior, and functional performance in applications ranging from detergents, personal care products, and pharmaceuticals, to food processing and petroleum recovery.^1–3^ Micellization is a fundamental process governing surfactant behavior, where these compounds assemble into micelles once their concentration exceeds a specific threshold. This threshold, referred to as the critical micelle concentration (CMC), represents a fundamental concept in colloid and interface science. The CMC does not only mark the beginning of micelle formation but also plays a decisive role in determining the overall efficiency of surfactant-based systems.^4,5^ Surfactants are widely used in both industrial and consumer applications. However, traditional measurement approaches present several limitations, including long testing times, limited experimental accuracy, and complex theoretical requirements. As a result, significant research efforts have focused on developing predictive models to estimate key physicochemical properties and link molecular structure to macroscopic performance.^6^ In this context, quantitative structure–property relationship (QSPR) models have been widely employed to predict surfactant properties. Such models establish mathematical correlations between molecular descriptors (inputs) and target properties (outputs), enabling the systematic identification of structure–property relationships. Molecular descriptors are numerical representations of chemical features derived from the molecular structure of a compound. They are computed according to defined mathematical rules implemented in specialized software, transforming structural information into quantifiable parameters suitable for modeling.^7,8^ Building upon this foundation, QSPR approaches can be combined with machine learning (ML) techniques to enhance predictive performance. These hybrid methods enable the identification of relevant molecular descriptors directly from data. Most QSPR studies in the surfactant field focus on predicting the CMC, although other key properties, such as surface tension at the CMC (ST_CMC_) and the hydrophile–lipophile balance (HLB), have also been investigated.^9–11^ With the enhanced computational capabilities now available, graph neural networks (GNNs) have emerged as promising tools for predicting surfactant properties.^12^ However, GNNs generally require large data sets to generalize effectively, whereas descriptor-based models perform robustly even with limited data. Moreover, the interpretability of molecular descriptors enables rational design, allowing the effects of structural modifications on predicted properties to be readily assessed.^13^

Surfactants are commonly classified according to the nature of their polar headgroup into ionic and nonionic types. Ionic surfactants dissociate in aqueous solution and are further divided into anionic, cationic, and zwitterionic species. Although zwitterionic surfactants contain both positive and negative charges, they are overall neutral and often exhibit behavior similar to nonionic systems. In contrast, nonionic surfactants do not ionize and generally show higher tolerance to pH and ionic strength, making them suitable for applications requiring mildness and stability.^14,15^ Furthermore, amphiphiles can also be classified based on their origin (biobased or petroleum-derived), or their molecular structure, such as conventional, bola type, or Gemini. Among these, Gemini surfactants, characterized by the presence of two polar head groups and two hydrophobic tails connected by a spacer at or near the head region, have attracted considerable attention due to their unique molecular architecture and superior interfacial properties.^16^ Despite their superior performance, including lower CMCs and higher surface activity compared to conventional single-headed surfactants, studies investigating structure–performance relationships and the design of novel Gemini surfactants remain relatively scarce. Most existing works have focused on predicting properties within a single surfactant class, typically constrained by structural similarities in headgroup charge or specific functional groups,^17^ with nonionic surfactants being the most extensively studied. This underscores the need for systematic approaches to understand and predict their behavior, complementing the extensive knowledge available for conventional surfactants.^18^

Numerous studies have shown that the structural features of amphiphiles govern a wide range of properties. In particular, the size and shape of the polar headgroup and hydrophobic tail play a critical role.^19^ In the context of surface tension reduction at the CMC (ST_CMC_), where it reaches its maximum effect, it is well established that ST_CMC_ tends to increase with larger polar head groups, while longer alkyl chains generally reduce ST_CMC_. Alkyl chain branching has a relatively minor effect, usually causing only a slight decrease in ST_CMC_.^20^ Recently, Flores et al.^21^ conducted a systematic study on sodium alkyl ethoxy sulfates, focusing on the separate effects of head and tail structures. They demonstrated that the hydrocarbon tail length predominantly controls micellar volume and viscosity, while the degree of ethoxylation of the polar head modulates the salt concentration required to reach peak viscosity. Qin et al.,^22^ further emphasized that CMC values are strongly influenced by molecular characteristics, including hydrophobic tail length and hydrophilic head area, but they also note that such qualitative relationships cannot be directly translated into quantitative predictions, thereby limiting the rational design and screening of surfactants. However, several empirical and QSPR approaches have already attempted to predict surfactant properties by considering the molecular characteristics of the polar and nonpolar regions separately. It is well established that the logarithm of the CMC decreases linearly with increasing hydrophobic chain length, forming the basis of the Stauff–Klevens equation.^23^ This behavior reflects the growing hydrophobic character of the tail with additional alkyl groups, which drives micelle formation at lower surfactant concentrations. However, these relationships are limited to the specific homologous series for which they were derived and for structurally simple surfactants.^24–26^ In line with this approach, Roberts^27^ developed a simple correlation for anionic surfactants, including primary alcohol sulfates and ester sulfates, relating the CMC to two parameters: the octanol/water partition coefficient (logP) of the hydrophobic moiety, defined as the logP of the entire molecule minus that of the negatively charged fragment, and the hydrophobic chain length, expressed as the number of C–C bonds. The works of Huibers et al.,^28^ Mahmoud Gad^29^ and Katritzky et al.^30^ are also noteworthy, as they employ fragment-based descriptors calculated independently for the polar head and the alkyl chain. These models, however, were restricted to nonionic surfactants, and the head–tail separation was defined simply as the region before the first heteroatom, without accounting for more complex structural variations. Wang and co-workers performed QSPR analysis for both nonionic^11^ and anionic^31^ surfactants to predict surface tension using three fragment-based descriptors. For nonionics, they considered the number of oxygen atoms in the hydrophilic segment, zero-order Kier and Hall index of the hydrophobic segment (KH_0_) and the heat of formation of a mole of surfactant molecule ΔH_f_. For anionic surfactants, the descriptors were KH_0_, ΔH_f_, and the molecular dipole moment (D), reflecting differences in how headgroup polarity is captured for each surfactant class. Similarly, Gaudin et al.^20,32^ developed QSPR models for sugar-based surfactants, predicting both CMC and ST_CMC_, in which fragment-based descriptors were computed separately for the polar head and the alkyl chain. Their models combined more than 300 constitutional, topological, geometrical and quantum-chemical descriptors were computed using for each whole surfactant and each fragment, computing almost 1000 descriptors calculated per molecule. These studies demonstrate the potential of separating polar and nonpolar regions to generate chemically meaningful descriptors. At the same time, they highlight the need for a more general and automated framework capable of handling a broader range of surfactant architectures. The separation of surfactant molecules into hydrophilic and hydrophobic components has traditionally relied on manual inspection, which can be time-consuming and prone to inconsistencies, particularly for complex or novel molecules. Domain-specific representations are widely used in current studies. For instance, Rother and Sadowski^33^ recently applied hetero segmented PC SAFT representing surfactants as assemblies of distinct groups, with separate parametrization for the hydrophobic tail and the hydrophilic head. Their work highlights the value of such domain-specific representations for both thermodynamic modeling and structure–property analysis. To the best of the authors’ knowledge, no method currently exists to automatically partition surfactant molecules into head and tail regions in a way that is (i) fully automated, (ii) computationally efficient, and (iii) sufficiently flexible to handle diverse surfactant classes and molecular architectures. Such a capability is essential for computational molecular design workflows, where rapid evaluation of hundreds or even thousands of candidate molecules is required. In this context, we propose an algorithm capable of automatically decomposing surfactant molecules into their hydrophilic and hydrophobic domains and computing specific structural descriptors for each domain. This approach minimizes human bias and ensures consistency across data sets. Unlike previous methodologies limited to conventional and structurally simple surfactants, the proposed framework can handle a wide range of molecular architectures, including branched, aromatic, Gemini, bola-type (two polar head groups located at opposite ends of a nonpolar tail), and other nonclassical structures. By explicitly characterizing each molecular domain such as head area, tail length and branching index, the method enables the construction of targeted predictive models with low computational cost, while improving interpretability and enhancing model extrapolation across surfactant classes. Furthermore, this domain-level representation enables the independent clustering of head and tail structures and the subsequent exploration of their cross-interactions, providing deeper insight into structure–property relationships and revealing potential synergistic or antagonistic effects arising from specific head–tail combinations. When integrated into multiobjective optimization frameworks, this approach further supports the rational and sustainable design of next-generation surfactant formulations.

In the remainder of this paper, we first present the decomposition-based methodology for surfactant property prediction in detail, starting with the automated division of each surfactant molecule into its hydrophilic and hydrophobic components. The following section describes the derivation of novel domain-specific descriptors that capture the structural features of heads and tails. These descriptors are then employed for both clustering analyses, aimed at identifying structural families and head–tail interaction patterns, and the development of predictive models for key surfactant properties. Subsequently, the performance of the proposed models is evaluated in the Results section. Finally, some conclusions are drawn.

## Methodology

The proposed framework overcomes key challenges in surfactant property prediction and design by systematically decomposing each molecule into hydrophilic head and hydrophobic tail domains using an automated molecular partitioning algorithm. As presented in Figure 1, each surfactant is represented by its SMILES notation, and the algorithm combines graph-theoretical operations with atom-level information such as atomic type and partial charges to generate chemically meaningful fragments. This decomposition enables the direct calculation of domain-specific descriptors, such as head area or tail branching, providing a robust and chemically grounded foundation for data-driven predictive modeling and rational design of surfactant systems.

**Figure 1:**
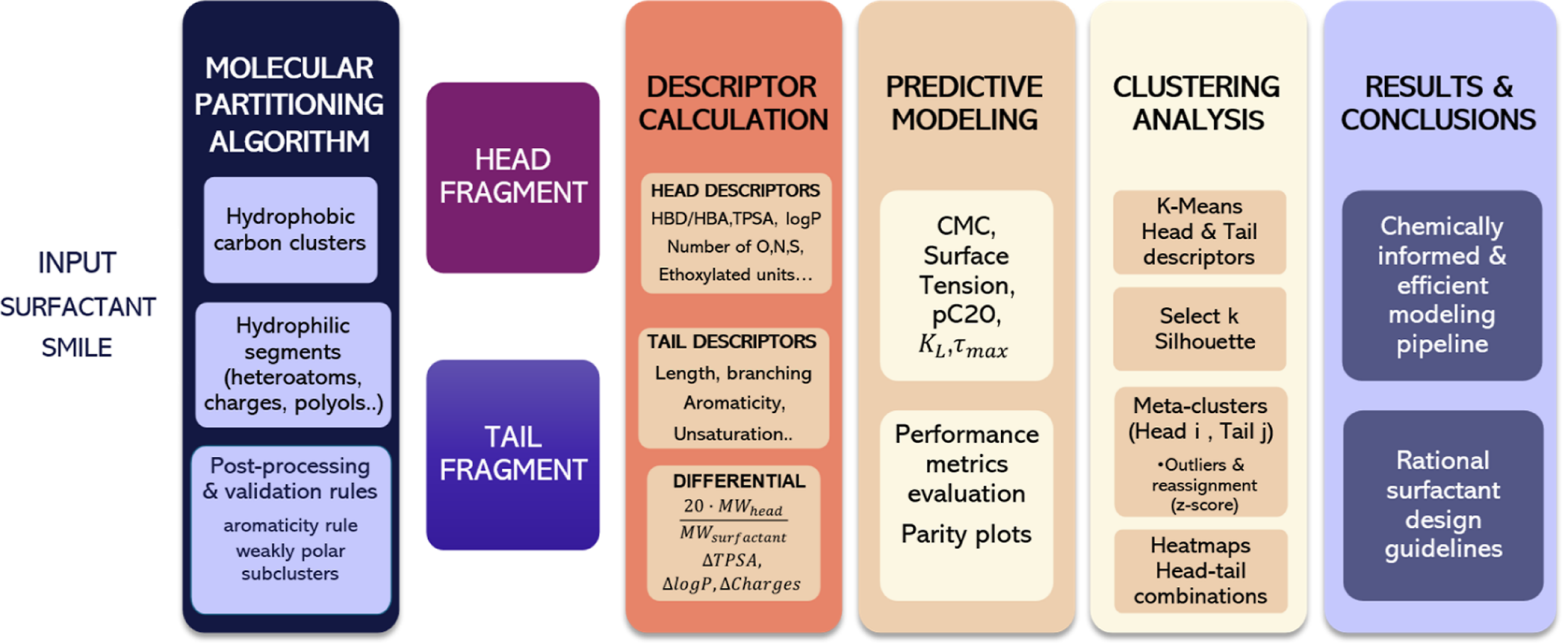
Workflow of the proposed automated Hydrophilic–Hydrophobic partitioning framework.

### Molecular Partitioning Algorithm

To quantitatively describe the amphiphilic nature of surfactant molecules and extract structure-based descriptors of their hydrophilic and hydrophobic regions, a molecular partitioning algorithm was developed. Some features are computed using RDKit descriptors module, an open-source cheminformatics package.^34^ Each surfactant molecule is represented by its SMILES notation, which encodes structural information about atoms, bonds, rings, aromaticity, and branching patterns. The algorithm systematically divides each molecule into chemically meaningful head (hydrophilic) and tail (hydrophobic) regions based on its topological and electronic structure, including some adjustable thresholds, as will be described later. This procedure ensures internal consistency and reproducibility across a broad range of surfactant types, including linear, branched, aromatic, and multitail structures. By automating the partitioning process, this method overcomes the subjectivity of manual classification, providing a standardized computational approach that can be applied to large data sets. The approach combines graph-theoretical operations with atom-level descriptors (atomic type, charge, hybridization, aromaticity, and atomic environment) to generate reproducible molecular fragments suitable for quantitative analysis.

### Identification of Hydrophobic Segments

Hydrophobic regions are detected as contiguous carbon clusters. The algorithm identifies all connected subgraphs composed exclusively of carbon atoms and their bonded hydrogens. The principal hydrophobic tail is predefined as the cluster with the largest number of carbons. Additional clusters are classified as secondary tails, which captures the structural features of Gemini or other multiple-tail surfactants. These secondary clusters must contain at least four carbon atoms to ensure that those represent genuinely hydrophobic segments rather than short alkyl substituents. This threshold is consistent with established physicochemical criteria distinguishing surfactants from hydrotropes, where fragments shorter than approximately four carbon atoms lack sufficient hydrophobic driving force for cooperative self-assembly.^35,36^ Small alkyl substituents bound to heteroatoms such as methyl, ethyl, or propyl groups, which are commonly found for instance on quaternary amines,^37,38^ are therefore not treated as independent hydrophobic segments. Instead, they are assigned to the headgroup domain, as their hydrophobic is minimal and their primary role is steric or electronic modulation of the polar head. This threshold can be adjusted if needed. Furthermore, chains containing halogenated carbons are evaluated for their hydrophobicity: perhalogenated chains, in which all carbons are bonded to halogen atoms (F, Cl, Br, I),^39^ are automatically classified as hydrophobic due to their low polarity and strong van der Waals interactions.

### Identification of the Hydrophilic Head

The hydrophilic head is initially seeded by atoms likely to be polar, including heteroatoms (O, N, S, P), atoms bearing formal charges and carbon atoms directly bonded to two or more heteroatoms, which typically belong to polar linkages (e.g., esters, amides, or polyether groups). Polyhydroxylated motifs, both (i) cyclic and (ii) open chains, are also incorporated into the head region owing to their intrinsically hydrophilic nature.

(i) Carbohydrate-like rings. Rings with a high oxygen density are treated as polar units. The algorithm evaluates each ring by counting heteroatoms within the cycle and exocyclic oxygen substituents directly attached to ring atoms. Rings containing three or more oxygen atoms in total are classified as carbohydrate-like. When such a motif is detected, the entire ring along with all oxygen-bearing substituents attached to it (i.e., including hydroxyl, anomeric, and short hydroxymethyl groups) is reassigned to the hydrophilic head.
(ii) Polyhydroxylated open chains. Linear or branched aliphatic chains enriched in hydroxyl substituents are similarly classified as part of the hydrophilic head. All carbons in the chain and their associated oxygen atoms are incorporated into the hydrophilic head, ensuring that partially oxidized or sparsely functionalized chains are not misclassified.

To refine the boundary between the hydrophilic and hydrophobic regions, a charge-based adjustment is performed. Partial atomic charges are calculated by RDKit using the Gasteiger–Marsili algorithm.^40^ Although these charges do not explicitly account for 3D electronic structure or solvation effects and are therefore less accurate than quantum-chemical methods (e.g., Hirshfeld or NPA^41^), they provide a computationally efficient and reproducible approximation of charge distribution. In this context, the objective is not to obtain absolute electronic accuracy, but to capture relative polarity trends across molecular regions. This enables consistent characterization of head–tail charge contrast and scalable processing of large surfactant data sets of hundreds to thousands of molecules. Gasteiger charges are widely used in the surfactant literature to describe charge distribution across structurally diverse surfactants.^42–46^ Atoms located at the interface between head and tail with an absolute charge magnitude |*q*| ≥ 0.25 are reassigned to the hydrophilic head. This correction, which can be modified as desired, aims to prevent underestimation of polar contributions near ester, amide, or ether junctions and ensures that regions contributing to hydrogen bonding and dipole–dipole interactions are correctly classified. Counterions, when present, are excluded from the headgroup to prevent errors in the subsequent charge analysis.

### Postprocessing and Validation Rules

To guarantee chemical and structural consistency, (i) aromaticity rule and (ii) reclassification of weakly polar subclusters are applied after the initial partitioning step.

Aromatic systems are characterized by delocalized π-electrons extending across the entire ring, which imparts uniform electronic properties and significant resonance stabilization. Partitioning such rings on an atom-by-atom basis would disrupt this electronic delocalization and lead to a chemically unrealistic representation. Therefore, in our algorithm, each aromatic ring is treated as an indivisible fragment. The ring is assigned entirely to either the hydrophobic or the hydrophilic region, depending on its chemical environment. Furthermore, benzene rings directly connected to hydrophobic carbon chains are always incorporated into the tail.^47^ This hierarchical identification approach ensures that the hydrophobic domain corresponds to the largest, least polar, and topologically continuous region of the molecule. This treatment ensures realistic behavior for aromatic-containing surfactants such as alkylbenzenesulfonates, phenol ethoxylates, and cationic pyridinium surfactants, where aromatic units may enhance either hydrophobicity or polarity depending on their substitution pattern.

To ensure a chemically consistent separation between the hydrophilic head and hydrophobic tail domains, an additional correction step was introduced to reclassify early weakly polar subclusters that are topologically disconnected from the dominant polar region by a neutral carbon bridge. This procedure aims to prevent mildly polar moieties, such as amide or carbonyl groups embedded within the hydrophobic chain, from being erroneously assigned to the head domain. When multiple disconnected polar fragments (head fragments) were detected within the initially identified head region, the algorithm first computed local descriptors for each fragment, including (i) the sum of absolute Gasteiger charges, (ii) the net formal charge, and (iii) the number of heteroatoms excluding counterions. The dominant polar fragment was then identified by prioritizing fragments with nonzero formal charge, followed by higher heteroatom counts and greater cumulative absolute charge. All remaining fragments were considered for reclassification as tail segments when the total charge magnitude of the dominant fragment was at least twice that of the candidate fragment, corresponding to a charge-ratio threshold of 2. This correction specifically targets betaine-type surfactants, where amide or carbonyl linkers form weakly polar subclusters. The threshold is consistently applied but only affects betaine-type structures; surfactants without ambiguous linkers remain unaffected, confirming that the criterion selectively corrects specific motifs without altering the data set globally. Following reassignment, head fragments are recomputed to preserve a consistent two-domain partitioning. The threshold is supported by observed charge ratios in representative betaine-family compounds, which range between 2.11 and 2.35 and can be adjusted if needed. An illustrative example of its application is provided in Algorithm validation section.

For each candidate, the shortest path between the candidate and the dominant fragment was computed within the molecular graph restricted to the head subgraph. If the connecting path contained a sequence of consecutive carbon atoms with low absolute Gasteiger charge, this path was interpreted as a neutral bridge. If the threshold is exceeded, the entire candidate subcluster, together with the neutral carbon bridge, was reclassified as part of the hydrophobic tail. The reassignment ensures that weakly polar moieties, when electronically and spatially insulated from the main hydrophilic domain, contribute to the tail rather than forming secondary artificial head groups. Following any reassignment, the head fragments were recomputed to update connectivity and preserve a consistent molecular partitioning. The molecular partitioning script can be found in the Github repository associated with this paper (see Data Statement for further details).

### Property Prediction

Explicit head and tail characterization can improve predictive modeling by generating feature representations that enhance interpretability and robustness while reducing computational cost and data pretreatment, ultimately improving extrapolation across different chemical families. In this way, chemically grounded domain separation contributes not only to molecular understanding but also to the development of data-efficient design frameworks for next-generation surfactants.

### Hydrophilic–lipophilic Balance (HLB) Estimation

In this work, Griffin’s method is used as a descriptor to reflect the proportion between the molecular weight of the hydrophilic head and the total molecular weight of the surfactant. While Griffin’s hydrophilic–lipophilic balance (HLB) was originally developed in 1949 for nonionic surfactants, particularly ethoxylated ones, it does not strictly apply to ionic or more structurally complex surfactants.^48^ Importantly, in our approach, HLB is not used as a quantitative measure of hydrophilic–lipophilic balance. Instead, it is used as a simple, generalizable descriptor to capture an essential structural feature of the molecule, the head-to-total molecular weight ratio. There are many methods to estimate HLB, built upon Griffin’s original concept, such as the group-contribution approach proposed by Davies,^49^ which provides more accurate HLB estimates by accounting for the contributions of individual hydrophilic and lipophilic groups, and more recent QSPR-based predictive models for different surfactants.^9,10^ However, our goal is not to calculate HLB precisely. The head-to-total molecular weight ratio serves as a computationally efficient and structurally meaningful descriptor, suitable for the development of predictive models of surfactant properties. This descriptor can influence critical properties such as micellization, interfacial activity, and aggregation behavior, providing a meaningful feature beyond the original empirical HLB context. HLB is calculated by the following eq 1, where MW refers to molecular weight.

1 $$HLB=20\times \frac{MW_{head}}{MW_{whole\;surfactant}}$$

The HLB scale ranges from 0 to 20, with low values indicating hydrophobic surfactants that favor solubilization in oils or W/O emulsions, intermediate values reflecting a balanced profile suitable for stable emulsions, and high values corresponding to strongly hydrophilic surfactants that promote water solubility and micelle formation.

### Descriptor Calculation

After partitioning, the algorithm outputs SMILES fragments corresponding to the hydrophilic head and hydrophobic tail(s). In this work, we propose a set of 24 domain-specific molecular features designed to capture the key structural and physicochemical elements governing surfactant behavior. Some features are computed using RDKit descriptors module, while others are derived from empirical relations or physicochemical calculations. This feature set combines intuitive molecular characteristics with computationally accessible parameters, ensuring interpretability while retaining predictive power.

The proposed features include Griffin’s HLB, tail chain length, tail volume, branching index, degree of unsaturation and aromaticity, the number of heteroatoms in the head (O, N, S), the number of ethoxylate units, formal headgroup charge, head area, hydrogen-bond donors (HBD) and acceptors (HBA), head and tail Topological Polar Surface Area (TPSA), ΔTPSA, head and tail logarithm of the octanol/water equilibrium partition ratio (logP), ΔlogP, and charge related terms.

Features associated with the hydrophilic head quantify its size, polarity, hydrogen-bonding capacity, and charge distribution, which are key determinants of solvation, headgroup repulsion, and interfacial packing. The head area is assessed by Labute’s Approximate Surface Area algorithm RDKit’s descriptor, that estimates the solvent-accessible surface area (SASA) of a molecule or fragment using molecular topology, without requiring a full 3D conformational optimization.^50^ Griffin’s HLB is used here as a descriptor of the relative contribution of the polar head to the overall molecule. Although it is not applied as a quantitative measure of hydrophilic–lipophilic balance for all surfactant classes, a larger relative contribution of the hydrophilic head is generally associated with greater hydrophilicity and higher CMCs, making this descriptor a meaningful and valuable feature for characterizing surfactant structure. Molecular weights are calculated using RDKit descriptors and eq 1 is computed. The number and type of heteroatoms (considering the most typical O, N, S) define potential sites for ionization and hydrogen bonding, while the number of hydrogen bond donors (HBD) and acceptors (HBA) quantify the molecule’s capacity to engage in such interactions. These descriptors were computed using the Lipinski module in RDKit. The number of ethoxylate units describes the repetition of oxyethylene chains, enhancing hydrophilicity and influencing aggregate curvature and morphology. Additionally, the TPSA and logP of the head fragment provide quantitative measures of polar surface area and hydrophilic–lipophilic balance, respectively, offering insight into solubility and interfacial orientation. RDKit descriptors are used for TPSA estimation and particularly Crippen module for logP estimation. Furthermore, two complementary charge-related features were derived to capture both macroscopic and microscopic aspects of the headgroup’s electronic structure using RDKit descriptors. The formal headgroup charge provides a discrete measure of the net ionic state, governing long-range electrostatic interactions, counterion binding, and surfactant classification (anionic, cationic, nonionic, or zwitterionic), which is also reported. In contrast, the charge-range feature, defined as the difference between the maximum and minimum Gasteiger partial charges within the fragment, captures intramolecular polarization and heterogeneity in electron density. While the formal charge reflects the overall electrostatic contribution, the charge-range feature describes local variations that influence solvation, hydrogen bonding, and interfacial orientation. These two features are therefore nonredundant and jointly provide a more complete representation of the electronic characteristics relevant to surfactant aggregation and interfacial behavior.

Features associated with the hydrophobic tail characterize the features that influence micellar packing, aggregation, and hydrophobic interactions. Tail length and volume provide fundamental measures of size, while structural properties such as unsaturation, branching, and aromaticity capture deviations from linearity and rigidity that affect packing efficiency and micelle curvature. It is important to note that chain length is described by two complementary features. The first is the number of atoms in the tail structure, while the second is the fully extended length of the hydrocarbon chain, calculated according to Tanford’s relation.^51^ Tail volume is assessed as Ghose–Crippen molar refractivity with Crippen module of RDKit, since molar refraction represents the hard-core volume of a molecule or atom.^52,53^ Aromatic rings introduce π–π stacking interactions, further modulating hydrophobicity, whereas TPSA and logP of the tail quantify residual polarity and lipophilicity, highlighting subtle differences in functionalized or partially polar tails. The charge-range feature is also computed for tail. To account for the interplay between the hydrophilic and hydrophobic domains, three differential descriptors were also defined. The difference in polar surface area (ΔTPSA) measures the polarity contrast between both fragments; larger values indicate stronger amphiphilic character and enhanced micellization tendency. The lipophilicity difference (ΔlogP) quantifies the hydrophobic driving force for aggregation. Finally, a differential charge-range feature (ΔChargeRange) was included to quantify the contrast in electronic polarization between the head and the tail. This feature is calculated from the spread of Gasteiger charges across both fragments to highlight asymmetries in electron distribution, which may be relevant in zwitterionic or pH-sensitive surfactants. The complete descriptor calculation script is available in the GitHub repository associated with this paper.

Overall, this descriptor set integrates both intuitive and computationally derived molecular features, providing a simple yet comprehensive representation of topology, polarity, and electronic distribution. Unlike traditional QSPR approaches that rely on hundreds or thousands of generic descriptors, this focused set emphasizes features with direct physicochemical meaning relevant to amphiphilic systems. Its simplicity minimizes redundancy, reduces overfitting, and enhances model generalizability. By separately accounting for head and tail contributions, the approach preserves the molecular logic underlying surfactant behavior, resulting in models that are not only accurate but also chemically interpretable and physically meaningful, offering clear insight into how molecular structure governs macroscopic properties such as critical micelle concentration and interfacial activity. Importantly, the proposed descriptor set remains flexible and can be adapted as needed.

### Surfactant Properties

The key properties considered in this work include the CMC, surface tension, adsorption efficiency (pC_20_), and interfacial adsorption kinetics, represented by maximum surface excess concentration τ_max_ and Langmuir constant, which are described in more detail in the following section.

The CMC is a key property in the design and evaluation of surfactants. It denotes the concentration at which surfactant molecules begin to self-assemble into micelles in solution. Below this threshold, the molecules remain predominantly as monomers, whereas above it, they spontaneously organize into micellar structures. The CMC also corresponds to the concentration at which the surface tension of the solution reaches a minimum.

Surface tension itself is another essential property governing surfactant behavior at liquid–gas interfaces. Surfactant molecules adsorb at the liquid–air interface, where they decrease the cohesive forces among liquid molecules, leading to a reduction in surface tension. This decrease facilitates improved spreading and wetting performance, enabling the surfactant to distribute more effectively across surfaces. To describe how surface tension decreases as surfactant concentration increases, the Szyszkowski equation is commonly employed.^54^ This semiempirical adsorption model relates the equilibrium surface tension of a solution to the bulk surfactant concentration under the assumption of a Langmuir-type adsorption isotherm. Therefore, in this equation two surfactant-specific parameters must be determined, the maximum surface excess concentration (τ_max_) and the Langmuir constant (*K*_L_). τ_max_ represents the maximum amount of surfactant that can be adsorbed at the interface, reflecting the interfacial packing limit while *K*_L_ quantifies the affinity of the surfactant for the interface, describing how quickly adsorption increases with concentration.

The adsorption efficiency (pC_20_) quantifies the concentration of surfactant necessary to achieve a specific degree of adsorption at the interface, defined as the negative logarithm of the surfactant concentration required to reduce interfacial tension by 20 mN/m. From surface tension versus logCMC curves, a 20 mN/m decrease in interfacial tension typically indicates that the interface of the surfactant solution is approaching saturation, making pC_20_ an optimal measure of adsorption efficiency and a practical metric for comparing the interfacial activity of different surfactants.^55,56^

### Clustering-Based Analysis of Head–Tail Interactions

To facilitate the analysis of head–tail interactions and uncover general structure–property relationships, head and tail structures were grouped into chemically meaningful sets using clustering techniques. Clustering, an unsupervised learning approach, uncovers hidden patterns within a data set by grouping similar objects together. These clusters enable a rapid, visual assessment of how different head and tail combinations influence the target property. By identifying trends within these clusters, this approach provides insights that can guide the rational design of new surfactants with tailored properties. Among the various clustering techniques, *K*-means is one of the most widely used and conceptually simple methods. The algorithm begins by randomly selecting initial centroids, which represent the centers of the molecular clusters. Each molecule is then assigned to the cluster whose centroid is closest to it. Through an iterative process, *K*-means alternates between two main steps: assigning molecules to clusters according to the current centroid positions, and updating the centroids based on the new cluster memberships. The similarity between molecules and centroids is typically evaluated using the Euclidean distance. The iterations continue until the centroids stabilize or a predefined number of iterations is reached.^57^ Determining the optimal number of clusters (*k*) for a data set is not a straightforward task, since there is no universal criterion that guarantees the best partition for all types of data. The most appropriate number of clusters often depends on the structure and distribution of the data itself. In this work, *k* was determined based on the Silhouette method. This approach evaluates how well each data point fits within its assigned cluster compared to other clusters. The Silhouette Index ranges from −1 to 1, where higher Silhouette value reflects stronger cohesion within clusters and greater separation between them.^58^ The optimal number of clusters can be selected as either the value of *k* that yields the highest average Silhouette score, or the smallest *k* at which the Silhouette values reach a plateau, suggesting that adding more clusters no longer improves the quality of the partition. The complete clustering workflow and implementation scripts can be found in the Github repository associated with this paper.

Building on the rationale introduced in the previous section where the limitations of relying on thousands of generic descriptors for modeling surfactant behavior where highlighted, the same considerations become even more critical in the context of unsupervised clustering. The use of more than a thousand descriptor clustering is particularly unsuitable due to the extreme redundancy, strong correlations, and large fraction of features with no physicochemical relevance to amphiphilic systems. In high-dimensional spaces, these issues amplify the curse of dimensionality, making distance metrics less informative and leading to unstable or chemically meaningless clusters. Employing the full descriptor set would require extensive preprocessing such as principal component analysis (PCA) or Pearson-based feature filtering, which removes features statistically and increases computational cost. In contrast, the domain-specific descriptors proposed in this work directly encode the structural elements most relevant to surfactant behavior. This fragment-wise, low-dimensional representation preserves interpretability, enables robust and chemically meaningful clustering for each surfactant region, and allows a precise analysis of head and tail specific structure–property relationships, all while greatly reducing computational cost.

## Results and Discussion

### Algorithm Validation

As an illustrative case, lauramidopropylbetaine (CCCCCCCCCCCC­(–O)­NCCC­[N+]­(C)­(C)­CC­([O-])=O) was analyzed. The initial partitioning identified two disconnected polar fragments: a dominant betaine moiety and a weaker amide fragment. The latter exhibited a substantially lower cumulative absolute charge (0.87 vs 1.84, which corresponds to a ratio of 2.11) and was linked to the dominant region through neutral carbon atoms with low Gasteiger charge. Since the amide contained only mild heteroatoms (C=O and NH) and lacked strongly polar sites, it met the demotion criteria. The ethylformamide fragment (C­(–O)­NCC) was therefore reclassified as part of the hydrophobic tail. This adjustment preserves chemical consistency by preventing the amide group, electronically isolated by a neutral bridge, from being misidentified as a secondary polar head. The resulting division shown in Figure 2 is consistent with that presented by Badmus et al.^59^

**Figure 2:**
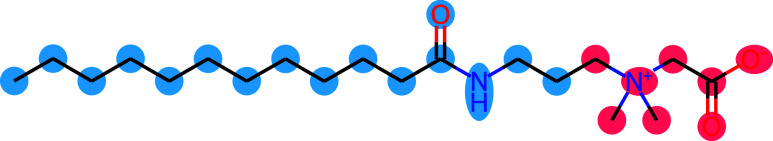
Hydrophilic head (red) and hydrophobic tail (blue) of lauramidopropylbetaine, as automatically identified using the proposed algorithm.

### HLB Calculation

To evaluate the performance and accuracy of the partitioning algorithm developed in this work, HLB values were calculated for a data set of 120 nonionic surfactants, comprising ethoxylated alcohols, ethoxylated fatty acid esters, ethoxylated dialkyl acids, and ethoxylated alkylphenols.^60^ These systems were selected because experimental HLB values are consistent with Griffin’s formulation only for nonionic surfactants, making them suitable for this purpose. In this context, HLB is used exclusively as a validation metric to assess whether the proposed algorithm correctly identifies and separates hydrophilic (head) and hydrophobic (tail) regions. This approach enables an assessment of the robustness of the partitioning algorithm independently of the broader applicability limitations of the HLB concept. The predictive framework achieved a high level of accuracy, with an *R* ^2^ of 0.94 and RMSE of 1.29 as shown in Figure 3. In addition, the automated identification of hydrophilic (head) and hydrophobic (tail) regions was successfully validated by comparison with manually assigned fragments for all compounds. Representative images of the identified head and tail regions are also available in the GitHub repository.

**Figure 3:**
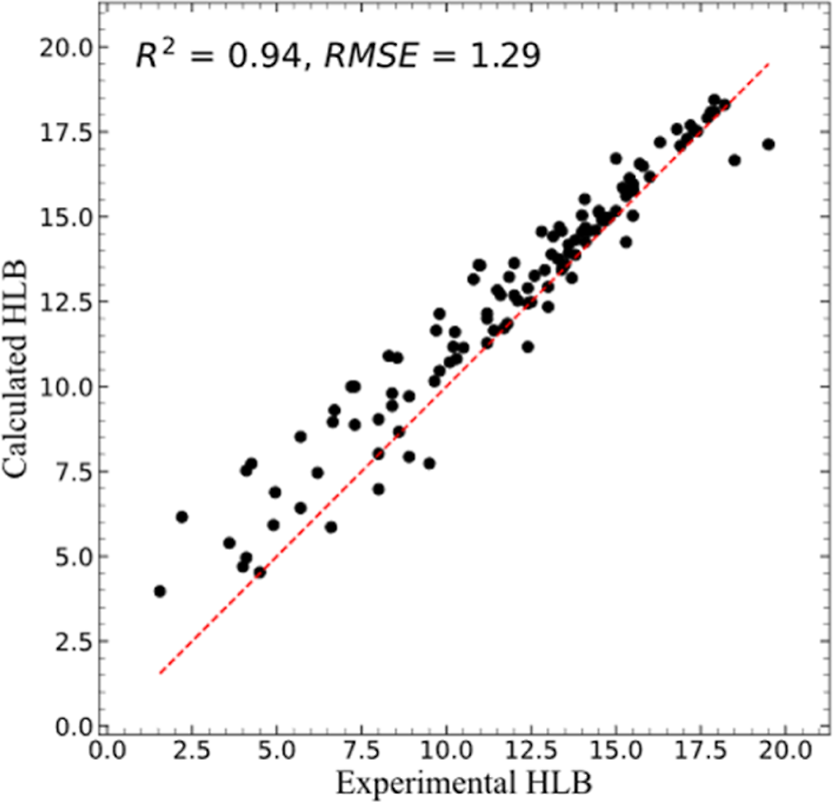
Parity plot comparing the calculated Griffin’s HLB values, obtained using the automated head–tail division, with the corresponding experimental measurements.

Overall, these results demonstrate the robustness and applicability of our automated partitioning framework. By accurately distinguishing head and tail regions across a diverse set of surfactants, the method provides a reliable foundation for the computation of domain-specific descriptors, enabling the efficient and physically informed prediction of additional surfactant properties.

### Property Prediction Models

Each database is randomly split according to the proportions reported in the respective original studies: 80/20 for all data sets, except for Qin et al.,^22^ which follows 90/10 split. Scikit-learn^61^ is used to appraise different modeling approaches, using functionality for data transformation, supervised learning, and model evaluation and selection. The mathematical form of each property model is chosen to achieve good balance between accuracy and statistical significance, with as simple a mathematical form as possible. Nonetheless, adsorption efficiency was modeled in accordance with the original publication for comparison, as the authors made the full set of hyperparameters available.

- Log­(CMC): Two databases are used to build models to predict critical micelle concentrations. Qin et al. gathered experimental CMC data for 202 surfactants, including 122 nonionic surfactants, 35 cationic surfactants, 34 anionic surfactants, and 11 zwitterionic surfactants. In this work, eXtreme Gradient Boosting (XGBoost) is employed to build the model with a maximum tree depth of 5 and gamma 0.1. Furthermore, Seddon et al.^62^ published CMC data for 91 surfactants and Partial least-squares regression (PLS regression) is applied with 12 principal components (PCs).
- Surface tension: In the same study by Seddon et al., surface tension data were compiled for the set of 91 surfactants. Their methodology involves independently predicting the Langmuir constant, the CMC, and the maximum surface excess concentration (τ_max_) using different sets of molecular descriptors and subsequently substituting these predicted values into the Szyszkowski equation to estimate surface tension.^54^ Importantly, Seddon et al. were the first to explicitly correlate these interfacial properties with molecular descriptors, thereby providing a highly valuable data set for subsequent studies. PLS regression is applied with 16 PCs.
- Log­(KL): Data of Langmuir constant is available in the work of Seddon et al., for 154 surfactants hydrocarbon surfactants categorized into 44 ethoxylates, 31 sulfates, 20 alcohols, 14 carboxylates, 14 amides, 6 betaines, 6 sulfonates, 5 tetra-alkyl ammonium surfactants, 4 glucosides, 4 pyrrolidinones, 3 pyridinium based surfactants and 3 glyceryl based surfactants. PLS regression is applied with 13 PCs.
- Maximum surface excess concentration (τ_max_): τ_max_ values for the whole set of 154 surfactants were likewise reported by Seddon et al. MLPRegressor architecture consisted of two hidden layers comprising 8 and 3 neurons, respectively, is applied. The hyperbolic tangent (tanh) activation function was selected and a learning rate of 0.1.
- Adsorption efficiency (pC_20_): Li et al.^63^ reported a pC_20_ data set comprising 124 surfactants, including complex quaternary ammonium salt Gemini surfactants, anionic Gemini surfactants with amide groups and long-chain polyethoxylated surfactants. According to the authors, the MLPRegressor used in their study consisted of two hidden layers with 50 neurons each. They further stated that the optimal hyperparameter combination was obtained using the relu activation function and the lbfgs solver. Our model employs the same architecture.

The performance metrics for all models are reported in Table 1 and parity plots shown in Figure 4.

**Table 1:** Metrics for the Property Prediction Models^a^

	data set	*R* ^2^_test_	RMSE_test_	*R* ^2^_train_	RSME_train_
log(CMC [μM])	202	0.87	0.36	0.99	0.14
log(CMC [M])	91	0.85	0.42	0.96	0.26
surface tension [mN/m]	91	0.75	2.96	0.92	1.79
log(*K*_L_[m^3^/mol])	154	0.82	0.68	0.85	0.64
log(τ_max_[mol/m^2^])	154	0.60	0.11	0.86	0.08
pC_20_log(C_20_[M])	124	0.94	0.34	0.99	0.10

a The size of the dataset denotes the total number of training and testing data. RMSE_*x*_ denotes the root mean squared error for the training (*x* = train) and testing (*x* = test) sets and carries the same units as the property of interest. Similarly, *R*_*x*_^2^ denotes the coefficient of determination for the training and testing sets, and it is dimensionless.

**Figure 4:**
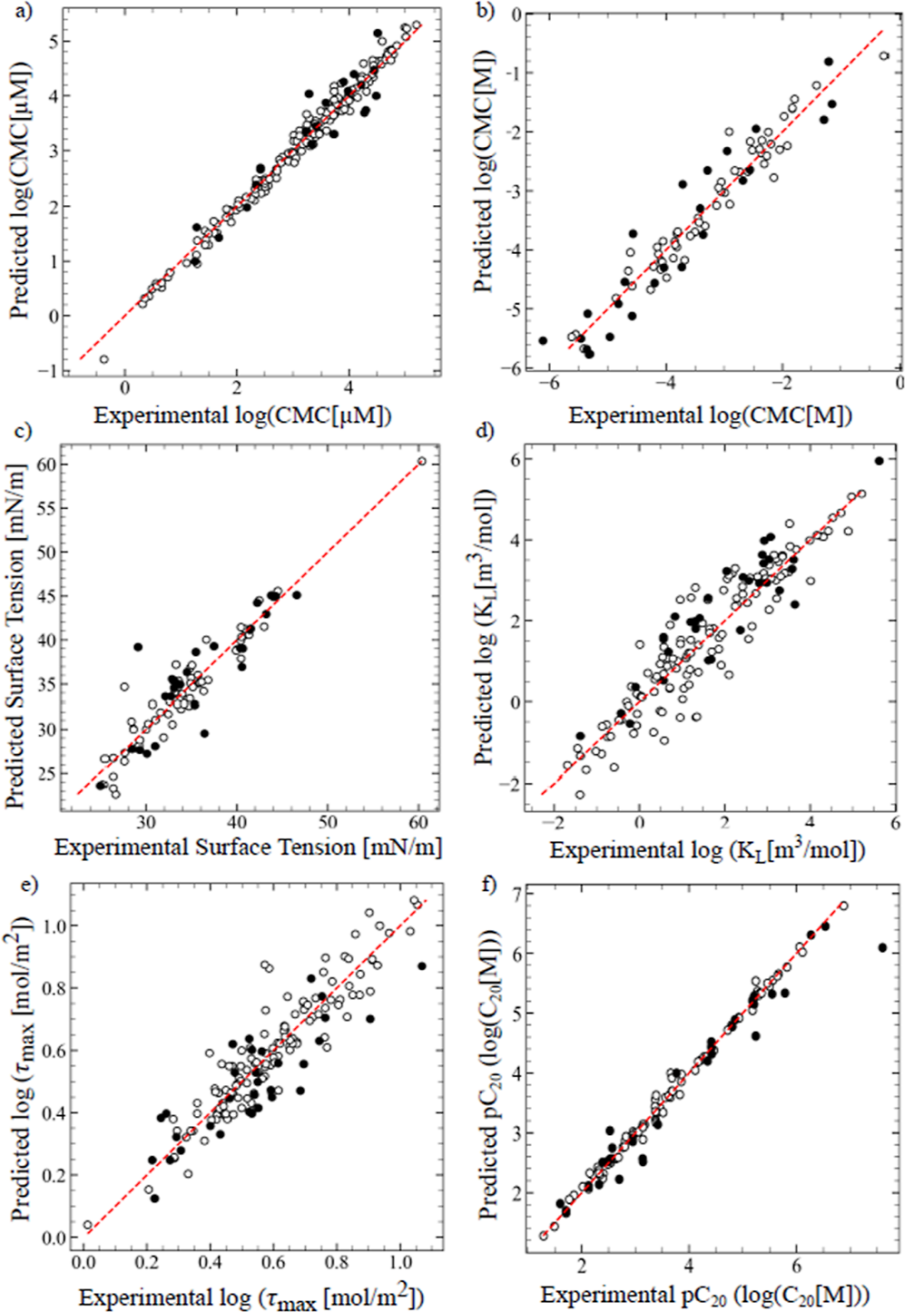
Parity plots showing the predicted values of the relevant properties vs the corresponding experimental values for (a) log­(CMC), where CMC is in μM, (b) log­(CMC), where CMC is in M, (c) surface tension, (d) log­(K_L_),where K_L_ is in m^3^/mol, (e) log­(τ_max_), where τ_max_ is in mol/m^2^, and (f) pC20 predictive models. The empty circles represent the training set, and the filled circles represent the test set.

Several models are built using data expressed on a logarithmic scale, a standard practice in QSPR preprocessing since the magnitude of many molecular properties can span multiple orders across different compounds.

As shown in Table 1, our results are highly comparable to those reported in the original publications. Qin et al. achieved *R* ^2^ of 0.92 and RMSE of 0.30 for test set with a Graph Convolution Network architecture. Seddon et al. reported *R* ^2^ values of 0.91 (training) and 0.69 (test) for τ_max_ using AlvaDesc (3D) library, 0.99 (training) and 0.79 (test) for LogKL using the SIRMS (2D) library, and 0.996 (training) and 0.87 (test) for logCMC using the CDK (3D) descriptors. Our approach achieves good predictive performance with a substantially simpler and more streamlined workflow without incorporating any 3D descriptors and without relying on multiple descriptor packages or additional feature-filtering steps for each property. Finally, Li et al. achieved a *R* ^2^ of 0.99 (training) and 0.92 (test), being slightly improved by our approach.

### Clustering-Based Analysis of Head–Tail Interactions

#### CMC Analysis

Taking the CMC as the main property of interest, clustering analysis was applied to evaluate the proposed method using the data set reported by Qin et al. Each surfactant molecule was first decomposed into its hydrophilic head and hydrophobic tail domains. Then, a total of 24 structural descriptors were originally computed within the proposed framework; however, descriptors referring to the overall molecular structure (i.e., ΔTPSA, ΔlogP, Δcharge, and Griffin’s HLB) were excluded from the clustering analysis, as they represent differential properties between head and tail domains. Consequently, only the domain-specific descriptors were retained: 11 describing head features and 9 describing tail features. The method is applied in a range of 4–100 clusters and the corresponding results for both heads and tails are presented in Figure 5. Structural variability is greater in the hydrophilic heads than in the tails, expecting a higher number of head clusters. The minimum clusters were defined as 4 due to the typical classification of surfactants in terms of their formal charge.

**Figure 5:**
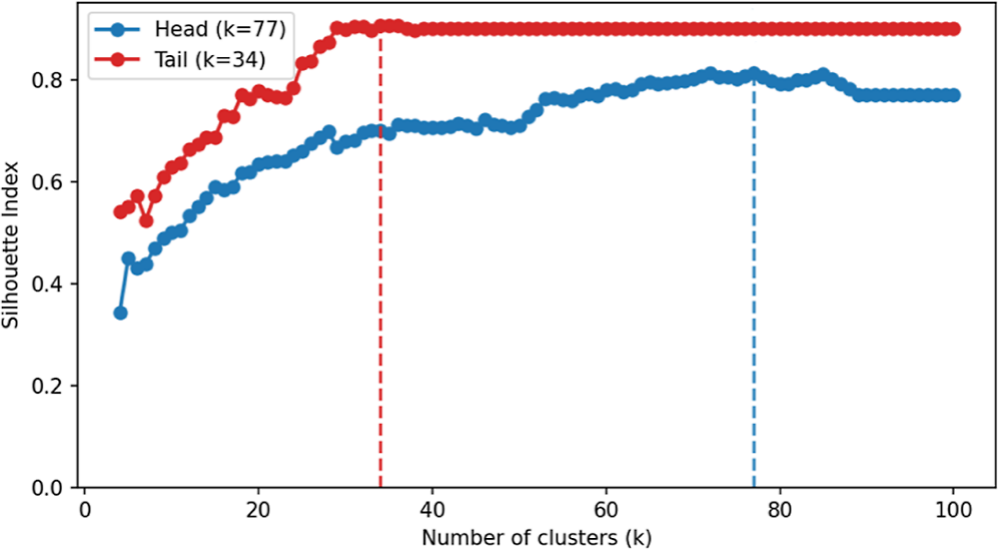
Silhouette Index as a function of the number of clusters (*k*) for the hydrophilic head (blue) and hydrophobic tail (red) regions identified using the proposed algorithm. Dashed vertical lines indicate the optimal number of clusters for each domain, corresponding to the highest silhouette score (*k* = 77 for head, *k* = 34 for tail).

The goal of this analysis is not to maximize clustering performance but to obtain a representative and chemically interpretable partition of head and tail structures. The aim is to identify general trends linking structural features of the hydrophilic head and hydrophobic tail to the target property. When an excessively large number of clusters is selected, as in Figure 5 where the maximum Silhouette Index values were obtained with 77 and 34 clusters for the head and tail domains, respectively, they reach very high values as 0.8–0.9. However, such high values indicate oversegmentation rather than meaningful structural differentiation. The data set becomes fragmented, clusters contain only a few structures, and the resulting averages lose statistical robustness and chemical interpretability. To avoid overfragmentation, a pragmatic silhouette-based criterion was adopted: the minimum number of clusters achieving an average silhouette coefficient of at least 0.6 was selected. This value is intended to ensure a suitable balance between compactness, separability, and interpretability, and is considered indicative of well-defined structures in molecular data sets characterized by high dimensionality and structural diversity.

Nonetheless, to ensure that any molecule deviates from the expected behavior of their structural clusters when analyzing the target property, an outlier-detection procedure based on meta-clusters. Each molecule was assigned to a head cluster and a tail cluster, and the pair was then treated as a single meta-cluster (i.e., h0-t0). Since the target property depends on the combined effect of both regions, analyzing outliers at this joint level allows us to capture deviations that cannot be detected from isolated structural partitions. Outliers were identified by evaluating the distribution of the target property within each meta-cluster using the z-score criterion. Molecules with anomalous values relative to their meta-cluster were flagged accordingly. For each meta-cluster, a centroid was computed, and a flagged molecule was reassigned if it was closer to the centroid of another meta-cluster and if this reassignment improved the agreement between its target value and the corresponding cluster distribution. If no valid reassignment was possible, this typically indicated that the tail clustering was too coarse. In such cases, the number of tail clusters were increased by one, and the full meta-clustering and outlier-detection process was repeated until reassignment is possible, or any outliers are detected. A schematic representation of the molecule reassignment process, based on z-score and centroid distance criteria, is included in the Supporting Information.

For this data set, any outlier was detected with the clusters assigned with 0.6 Silhouette score. The combination yielding the minimum log CMC corresponds to Cluster 13 in head and Cluster 4 in tails, as depicted in Figure 6. The Head Cluster 13 comprises ethoxylated structures with 4–7 EO units, while the Tail Cluster 4 includes linear alkyl chains ranging from 14 to 16 carbon atoms. The optimal combination is a head with 6 EO units and a tail with 16 carbon atoms belonging to this h13-t4 metacluster. Notably, Head Clusters 6 and 10 also consist of ethoxylated structures with 8–12 EO units and 15–21 EO units, respectively, corresponding to the next best CMC values. The same tail cluster can produce considerably higher CMC values when paired with Head Cluster 1, which includes aromatic quaternary ammonium heads, aliphatic tetraalkylammonium heads, and amino acid–derived zwitterionic heads (valine- and leucine-based). Interestingly, when Head Cluster 1 is combined with Tail Cluster 5, which consists of short linear alkyl chains ranging from 4 to 8 carbon atom, the resulting combination exhibits the highest and undesirable CMC values. Detailed cluster characterization is provided in the Supporting Information, including descriptor effect–size profiles (Figures S2 and S3) and representative structures for each cluster (Tables S2 and S3).

**Figure 6:**
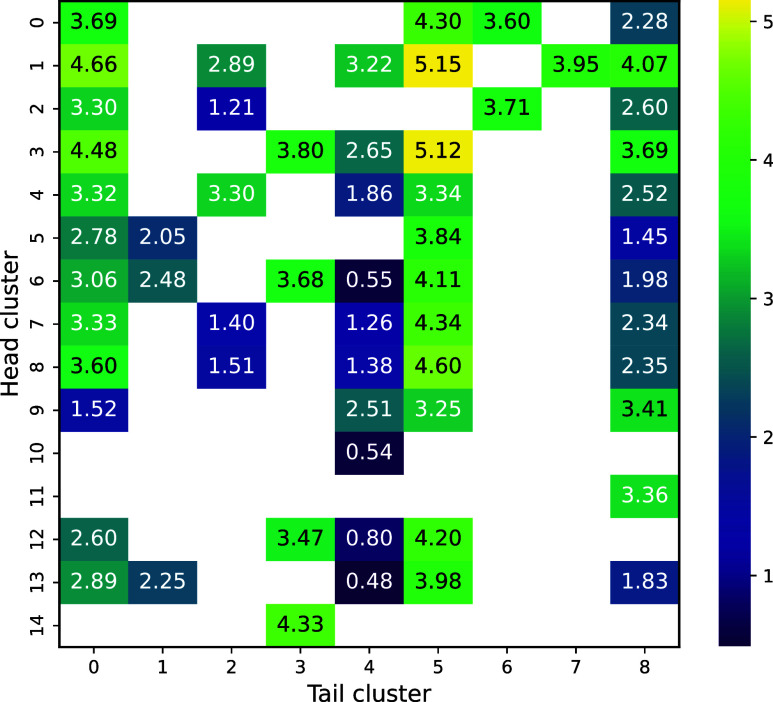
Heatmap showing the average logCMC, where CMC is in μM, of surfactants corresponding to each combination of headgroup cluster (*y*-axis) and tail cluster (*x*-axis). Color scale represents the magnitude of the average logCMC, with yellow-green indicating higher values, and blue-purple indicating lower values. Empty cells indicate combinations for which no surfactants were present in the data set.

#### pC20 Analysis

Clustering results for the pC_20_ data set are also analyzed to illustrate the markedly different structural diversity observed across databases. This contrast highlights both the difficulty of selecting an optimal number of clusters and the relevance of the proposed method, which adapts to changes in data set size and heterogeneity. Furthermore, in the original publication of this data set, the authors also performed clustering using the full molecular representation rather than separating structural regions, making this comparison particularly relevant.

For this new data set, the minimum number of tail clusters required to meet the silhouette score threshold of 0.6 was four, which is the smallest number considered. However, outliers were detected and could not be reassigned until the number of tail clusters was increased to six, as shown in Figure 7.

**Figure 7:**
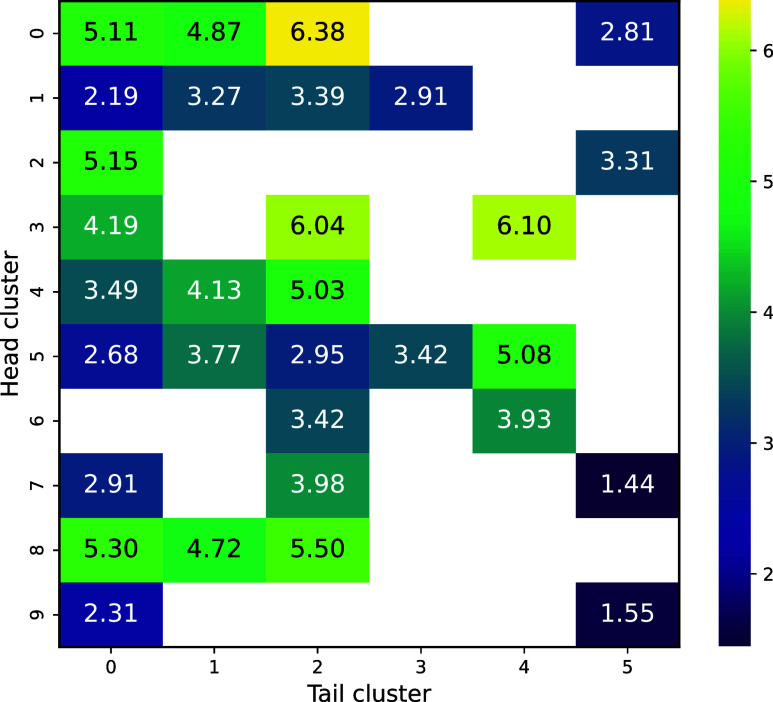
Heatmap showing the average pC20 of surfactants corresponding to each combination of headgroup cluster (*y*-axis) and tail cluster (*x*-axis). Color scale represents the magnitude of the average pC20, with yellow-green indicating higher values, and blue-purple indicating lower values. Empty cells indicate combinations for which no surfactants were present in the data set.

The highest adsorption efficiency values correspond to the combination of Cluster Head 0 and Cluster Tail 2, which feature ethoxylated heads with 4 to 9 EO units and linear alkyl chains of 14 to 18 carbons, respectively. It is worth noting that Cluster Head 8 also corresponds to ethoxylated headgroups but with 12 to 16 EO units, further confirming that smaller ethoxylated heads lead to higher pC_20_ values. In addition, when the same Tail Cluster is combined with Cluster Head 3, which contains glucosamide and bis-amide headgroups, high pC_20_ values are also observed. This effect is further enhanced when Cluster Head 3 is combined with Cluster Tail 4, which corresponds to long double-tails. In contrast, the lowest adsorption efficiency is observed for Cluster Tail 5, which comprises short alkyl chains of 6 to 9 carbons, when paired with the pyridine-based structures grouped in Cluster 7. These observations support the statement from the original paper, in which the authors reported that an increase in the number of carbon atoms in the alkyl chain generally leads to higher pC_20_ values. However, the present results provide a more detailed view by clustering head and tail groups separately, as even within the same tail cluster differences of up to 4.33 pC20 units emerge depending on the head cluster with which it is combined. Detailed cluster characterization is provided in the Supporting Information, including descriptor effect–size profiles (Figures S4 and S5) and representative structures for each cluster (Tables S4 and S5).

#### Surface Tension Analysis

Surface tension was also analyzed through clustering, showing that small polyol-based headgroups such as glycerol, ethylene glycol, and propanediol units exhibit the lowest values when combined with short alkyl tails of up to 10 carbon atoms. Detailed cluster characterization is provided in the Supporting Information, including descriptor effect–size profiles (Figures S6 and S7) and representative structures for each cluster (Tables S6 and S7).

Calculated molecular features and visual representations of the head–tail partitioning and clustering for each target property are provided in the GitHub repository associated with this paper.

## Conclusions

This work presents a novel methodology for predicting key surfactant properties by introducing an explicit, automated, and systematic head–tail molecular decomposition. Each molecule in the data set is partitioned into its hydrophilic head and hydrophobic tail, reducing human bias and the time-consuming manual segmentation traditionally required, while enabling the consistent characterization of complex or unconventional structures and the calculation of specialized descriptors tailored to each domain. This chemically informed representation significantly enhances the modeling pipeline by embedding structural knowledge directly into the feature space.

Using these domain-specific features, predictive models were developed to estimate fundamental surfactant properties, including the CMC, surface tension, maximum package, Langmuir constant, and pC_20_. Complementary clustering analyses were also performed to identify the most favorable combinations of head and tail structures, revealing which structural motifs most strongly enhance or hinder performance metrics. Together, these results establish a clearer understanding of how these combinations govern surfactant behavior and provide a foundation for rational surfactant design guided by structure–property relationships rather than purely empirical heuristics.

The proposed descriptors cover key aspects of surfactant structure, including tail size and topology, headgroup composition and charge, polarity and hydrogen-bonding capacity, and partitioning-related properties. The resulting feature representations strengthen model robustness, reduce data-driven artifacts, and enhance extrapolation across diverse chemical families. In this way, chemically grounded domain separation contributes not only to a deeper molecular understanding but also to the development of data-efficient and principled design frameworks for next-generation surfactants. Importantly, this framework has broad implications for industrial surfactant design. By automating the separation of hydrophilic heads and hydrophobic tails, it enables rapid, large-scale analysis of diverse surfactant libraries and facilitates the identification of optimal combinations for target properties. This systematic, reproducible, and interpretable approach can accelerate the development of high-performance surfactants while reducing reliance on trial-and-error experimentation. Furthermore, by incorporating features that capture the presence of pH, temperature or light-sensitive functional groups, the framework could be extended to predict dynamic self-assembly and property changes in stimuli-responsive systems. Such an extension would support the rational design of adaptive surfactants for industrial and biomedical applications, including controlled-release formulations, responsive emulsions, and smart surface-active materials. Furthermore, the method can be applied to biosurfactants, including those with complex head structures such as glycoproteins, polysaccharides, or lipid-based moieties. While the current feature set based on 2D molecular information is effective and computationally efficient, future extensions of the framework could incorporate additional descriptors to capture subtler steric and conformational effects. Possible additions could include McGowan volume, solvent-excluded surface (SES) mesh-derived metrics, or other 3D molecular descriptors. Such enhancements could improve accuracy for properties sensitive to molecular geometry, although they would come with higher computational cost and potentially require specialized algorithms or external software. Ultimately, a balance must be maintained between descriptor richness and computational tractability, depending on the intended application and the scale of the chemical space to be explored.

## Supplementary Material



## Data Availability

Code, results and models related to this article can be found on the Github repository https://github.com/SofiaGNu/High-Efficiency-Prediction-of-Surfactant-Properties-through-Automated-Head-Tail-Partitioning and are available under a CC BY 4.0 license.

## Notes

Further details are provided in the Supporting Information, including the proposed domain-specific descriptors and their physicochemical relevance (Table S1), the validation of Gasteiger charges against quantum-chemical methods (Figure S1), and the cluster interpretability analyses, including effect–size profiles (Figures S2–S7) and representative structures (Tables S2–S7), which support their chemical interpretation across CMC, pC20, and surface tension data sets. In addition, the explanation of the outlier detection and metacluster reassignment procedure has been included with a flowchart (Figure S8) to ensure clarity and reproducibility (PDF)
