# Supplementary material for: High-Efficiency Prediction of Surfactant Properties through Automated Hydrophilic–Hydrophobic Partitioning
Source: J Chem Inf Model. 2026 Jun 2;66(12):6933–45. doi: 10.1021/acs.jcim.6c00398 (PMC13292217; doi:10.1021/acs.jcim.6c00398)
Supplement: Supplementary file 1 [file ci6c00398_si_001.pdf]

High-Efficiency Prediction of Surfactant Properties through Automated Hydrophilic–Hydrophobic Partitioning

Sofía González-Núñez[a], Carlos Amador[b], Mariano Martín[*a1]

[a] Departamento de Ingeniería Química. Universidad de Salamanca. Pza. Caídos 1-5, 37008 Salamanca, Spain
[b] Newcastle Innovative Centre. Procter and Gamble. Whitley Rd, Longbenton, Newcastle Upon Tyne, Tyne And Wear NE12 9SR, England

DOMAIN-SPECIFIC MOLECULAR FEATURES PROPOSED

Table S1 summarizes the set of domain-specific molecular features designed to capture the key structural and physicochemical elements governing surfactant behavior. For each descriptor, a concise definition is provided, along with its physicochemical relevance, highlighting how it relates to surfactant behavior, micellization, and aggregate formation.

Table S1. Descriptor Definitions and Physicochemical Relevance

Descriptor	Definition	Physicochemical relevance
**Griffin's HLB**	$HLB = 20 \cdot \frac{MW_{head}}{MW_{whole\,surfactant}}$	Captures the relative contribution of the hydrophilic head. Influences micellization, interfacial activity, and aggregation behavior. Used as a structural descriptor rather than a quantitative HLB scale
**Tail chain length**	i) Number of atoms and ii) fully extended chain length (Tanford relation)	Governs micellar packing and aggregate size
**Tail volume**	Molecular volume estimated via molar refractivity (Crippen)	Related with hydrophobic volume and micelle formation
**Branching index**	Degree of branching in the hydrophobic chain	Affects packing efficiency and micelle curvature
**Degree of unsaturation**	Number of double bonds in the chain	Influences rigidity and packing behavior
**Aromaticity**	Presence of aromatic rings	Enables π–π interactions and modifies hydrophobicity
**Number of heteroatoms (O, N, S)**	Count of polar atoms in the headgroup	Determines ionization sites and interaction potential
**Number of ethoxylate units**	Number of –(OCH_2CH_2)– repeating units	Increases hydrophilicity and affects aggregate curvature

[1] M. Martín mariano.m3@usal.es

**Formal headgroup charge**	Net charge of the hydrophilic head	Governs electrostatic interactions and surfactant classification
**Headgroup area**	Solvent-accessible surface area (SASA, Labute method)	Influences interfacial packing and steric repulsion
**HBD (H-bond donors)**	Number of hydrogen bond donor groups	Controls intermolecular interactions and solvation
**HBA (H-bond acceptors)**	Number of hydrogen bond acceptor groups	Affects hydrogen bonding and solution stability
**TPSA (head/tail)**	Topological polar surface area of each fragment	Relates polarity to solubility and interfacial orientation
**ΔTPSA**	Difference in TPSA between head and tail	Measures polarity contrast. Larger values enhance amphiphilicity and micellization
**logP (head/tail)**	Octanol/water partition coefficient	Indicates hydrophilic–lipophilic balance
**ΔlogP**	Difference in logP between head and tail	Reflects hydrophobic driving force for aggregation
**Charge range (head/tail)**	Difference between maximum and minimum partial charges	Captures local electronic polarization
**ΔChargeRange**	Difference in charge range between head and tail	Describes electronic asymmetry relevant to aggregation

GASTEIGER–MARSILI CHARGES

Gasteiger charges are widely used in cheminformatics as topology-based approximations of partial atomic charges. These charges are computed purely from molecular topology and electronegativity equalization principles, without accounting for the full three-dimensional electronic structure or explicit solvation effects. As a result, they do not capture polarization or solvent interactions, which are important for a complete electrostatic description of amphiphilic molecules. In the present study, Gasteiger charges are employed as one of several complementary descriptors, alongside topological, connectivity, and chemical features as shown in Table S1.

More sophisticated quantum-chemical methods provide a more accurate description of partial atomic charges. However, these methods are computationally demanding for large datasets. Gasteiger charges offer a practical compromise, providing a consistent approximation of charge distribution that preserves the essential polarity contrasts between head and tail regions. To illustrate this, Gasteiger charges were compared with Hirshfeld and NPA charges reported for the cetylpyridinium cation. In the study by Fizer and Fizer[1], partial atomic charges were computed using Hartree–Fock and six DFT functionals (PBE, TPSS, B3LYP, PBE0, M06, and ωB97) and evaluated via CHELPG, Mulliken, Löwdin, Hirshfeld, and natural population schemes. The authors concluded that Hirshfeld and NPA charges are most suitable for modeling electrostatic interactions in systems containing the cetylpyridinium cation, although they noted that the nitrogen partial charge is highly sensitive to the computational method used. As shown in the Figure S1, we calculated Gasteiger charges and compared them with the Hirshfeld and NPA values reported by Fizer et al.

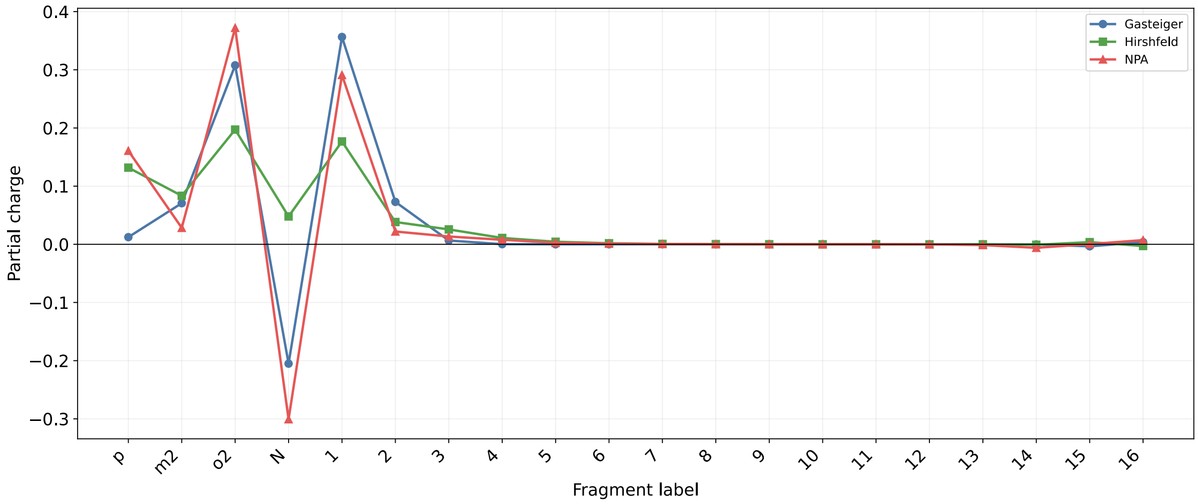

Figure S1. Comparison of partial atomic charges along the cetylpyridinium cation. Gasteiger charges are shown in blue, Hirshfeld charges in green, and NPA charges in red. The x-axis represents atom positions along the molecule: o, m, and p indicate the ortho, meta, and para positions of the pyridinium ring, while positions 1–16 correspond to the alkyl chain, numbered from the carbon attached to the nitrogen (1–15CH_2 and terminal 16CH_3). The y-axis represents the corresponding partial charges.

While absolute magnitudes differ, all methods display the same physically relevant trend: the charge is concentrated in the polar head region (positions O/N/1) and decays to near-zero along the alkyl tail (positions 4–16). Quantitatively, all methods show a strong head–tail contrast in mean absolute charge, and Gasteiger charges correlate strongly with NPA values. This comparison confirms that Gasteiger charges, despite being approximate, reliably capture relative charge distribution and polarity trends, supporting their use as a computationally efficient descriptor for scalable head–tail partitioning in large surfactant datasets.

CLUSTER INTERPRETABILITY ANALYSIS

To better understand the chemical meaning of the clusters obtained for surfactant heads and tails, a Cluster Interpretability Analysis was conducted. This analysis demonstrates that each cluster corresponds to a chemically coherent grouping rather than an arbitrary mathematical construct. For each cluster, two aspects were evaluated: (i) descriptor effect-size profiles, which highlight the key physicochemical properties that differentiate clusters, and (ii) representative medoid structures, i.e., the head or tail molecule that best represents the cluster by minimizing the average Euclidean distance to all other members. Combining these two approaches validates the chemical relevance of the clusters and provides a clear rationale for their use in guiding surfactant design, showing how structural motifs relate to the target property of interest.

For each cluster, the effect size of every numeric descriptor is defined as:

$$d_j^{(c)} = \frac{\grave{x}_j^{(c)} - \grave{x}_j^{\text{all}}}{\sigma_j^{\text{all}}}$$

where $\grave{x}_j^{(c)}$ is the within-cluster mean of descriptor j, $\grave{x}_j^{\text{all}}$ and σ_j^{all} are the global mean and standard deviation.

Effect size normalizes each descriptor to a common scale and quantifies how much a given structural feature is over- or under-represented in a cluster relative to the entire dataset. This formulation enables mechanistic interpretations, such as ethoxylated, sulfur-containing, or short-chain motifs, to be supported by quantitative evidence rather than qualitative inspection. Importantly, the dominant descriptors identified for each cluster correspond closely to the structural features of the representative structure, confirming that the clusters represent chemically coherent groupings rather than arbitrary numerical partitions in descriptor space.

Table S2: Representative Structures of Head Clusters in CMC Analysis

Cluster ID	Head
**0**	OCC1OC(O)C(O)C(O)C1O
**1**	C[N+](C)C
**2**	OCC1OC(OC2C(CO)OC(O)C(O)C2O)C(O)C(O)C1O
**3**	O=S(=O)([O-])O
**4**	C[N+](C)CC(=O)[O-]
**5**	OCCOCCO
**6**	OCCOCCOCCOCCOCCOCCOCCO
**7**	CNC(=O)C(O)C(O)C(OC1OC(CO)C(O)C(O)C1O)C(O)CO
**8**	O=CNC1OC(CO)C(O)C(O)C1O
**9**	O=C1OC(S)C(O)C1O
**10**	OCCOCCOCCOCCOCCOCCOCCOCCOCCOCCOCCOCCOCCO
**11**	O=COCC1OC(OCC2OC(OCC3OC(OC4(CO)OC(CO)C(O)C4O)C(O)C(O)C3O)C(O)C(O)C2O)C(O)C(O)C1O
**12**	OCC(O)C(O)C(O)CS
**13**	OCCOCCOCCOCCOCCOCCO
**14**	O=C[O-]

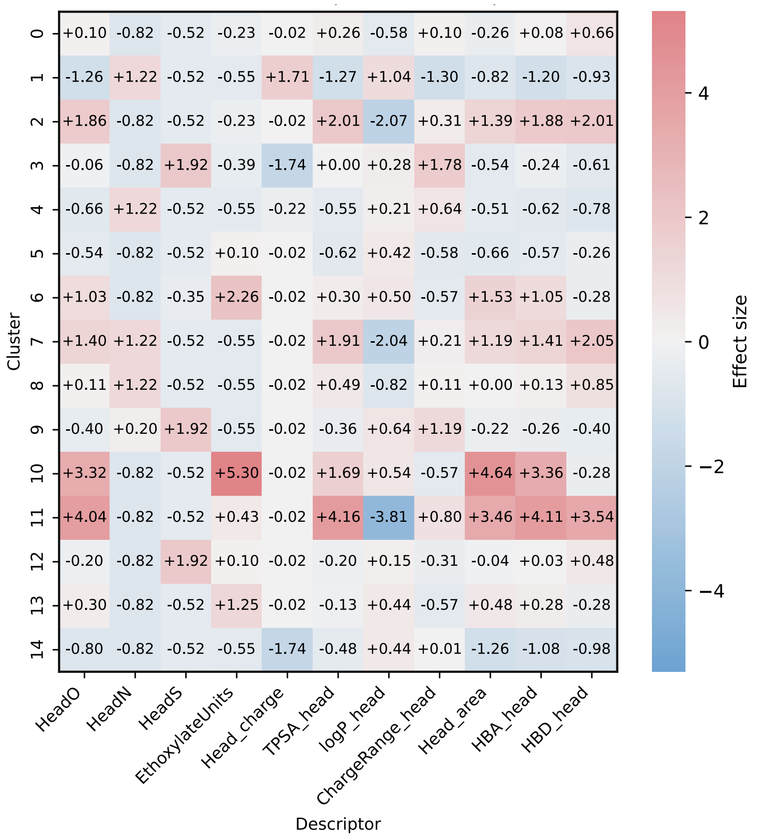

Figure S2. Descriptor effect-size profiles for head clusters in CMC Analysis. Heatmap showing the standardized effect size of each descriptor across clusters. Positive values (red) indicate that a descriptor is overrepresented within a given cluster relative to the full dataset, whereas negative values (blue) indicate underrepresentation. The magnitude of the effect size reflects the discriminative strength of each descriptor, highlighting the key physicochemical features that define and differentiate the structural characteristics of each cluster.

Chemical interpretation of head clusters (H0–H14)

- H0: polar, N/S-poor polyol-like heads (sugar/polyol medoid).
- H1: cationic ammonium-rich heads (high charge, low TPSA; trimethylammonium-like medoid).
- H2: highly oxygenated, strongly hydrophilic heads (high HBA/HBD; polysaccharide-like).
- H3: sulfur-containing anionic heads (sulfate/sulfonate signatures).
- H4: zwitterionic or amine–carboxylate-like heads.
- H5: small low-area ethoxylated heads with moderate polarity.
- H6: medium-EO ethoxylates (high EO, high head area).
- H7: very hydrophilic, strong hydrogen-bonding heads (amide/sugar-like).
- H8: polar amide/sugar-like heads with N-containing character.
- H9: sulfur-heterocycle-like heads with relatively higher hydrophobic character.
- H10: high-EO ethoxylates (very large oxygen-rich headgroups).
- H11: ultra-polar/high-oxygen head chemistry (extreme oxygenation profile).
- H12: sulfur-containing heads with intermediate hydrophilicity.
- H13: canonical ethoxylated family (mid EO, low charge), strongly associated with low CMC regimes.
- H14: very small/simple anionic heads (low structural complexity).

Table S3: Representative Structures of Tail Clusters in CMC Analysis

Cluster ID	Representative Tail
**0**	CCCCCCCCCC
**1**	CC(C)(C)CC(C)(C)c1ccccc1
**2**	CCCCCCCCC=CCCCCCCCC
**3**	CC(F)(F)C(F)(F)C(F)(F)C(F)(F)C(F)(F)C(F)(F)C(F)(F)F
**4**	CCCCCCCCCCCCCCC
**5**	CCCCCCCC
**6**	CCC(C)CCCC(C)C
**7**	CCCCCCCCCCCCCc1ccccc1
**8**	CCCCCCCCCCCC

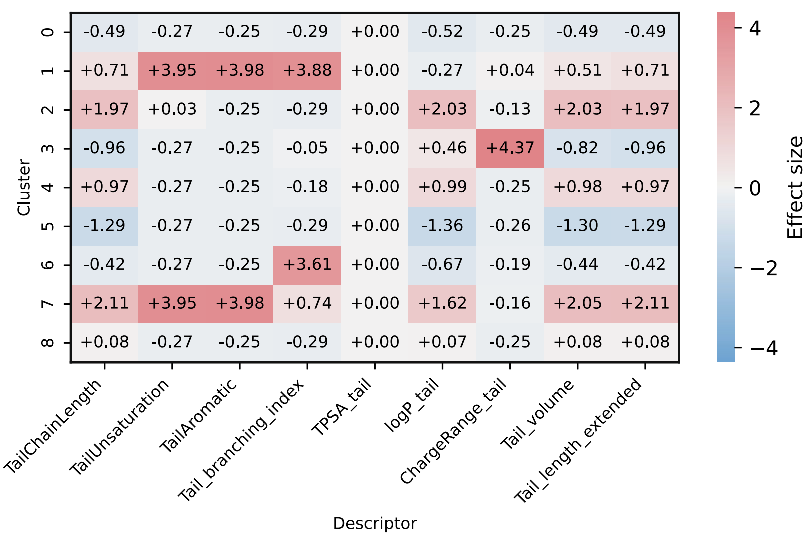

Figure S3. Descriptor effect-size profiles for tail clusters in CMC analysis. Heatmap showing the standardized effect size of each descriptor across clusters. Positive values (red) indicate that a descriptor is overrepresented within a given cluster relative to the full dataset, whereas negative values (blue) indicate underrepresentation. The magnitude of the effect size reflects the discriminative strength of each descriptor, highlighting the key physicochemical features that define and differentiate the structural characteristics of each cluster.

Chemical interpretation of tail clusters (T0–T8)

- T0: medium linear alkyl tails (approximately C10).
- T1: aromatic, unsaturated, branched tails.
- T2: very long and highly hydrophobic tails (including long unsaturated motifs).
- T3: tails with high charge-range dispersion (perfluorinated).
- T4: long linear hydrophobic tails (approximately C14–C16), optimal for low CMC when paired with ethoxylated heads.
- T5: short linear tails (approximately C4–C8), associated with high CMC.
- T6: branched aliphatic tails.
- T7: long aromatic/unsaturated tails.
- T8: intermediate linear saturated tails (C12 approx. baseline, low branching).

pC20 Analysis

Table S4: Representative Structures of Head Clusters in pC20 Analysis

Cluster ID	Representative Head
**0**	OCCOCCOCCOCCOCCOCCOCCO
**1**	O=S(=O)[O-]
**2**	OCCCO
**3**	CNC(=O)C(O)C(O)C(O)C(O)CO
**4**	C[N+]CC(=O)[O-]
**5**	C[N+](C)C.O=S(=O)([O-])O
**6**	C[N+](C)CC(O)COCCOCCOCC(O)C[N+](C)C
**7**	O=S(=O)([O-])OCCOCCO
**8**	OCCOCCOCCOCCOCCOCCOCCOCCOCCOCCOCCO
**9**	C[n+]1ccccc1

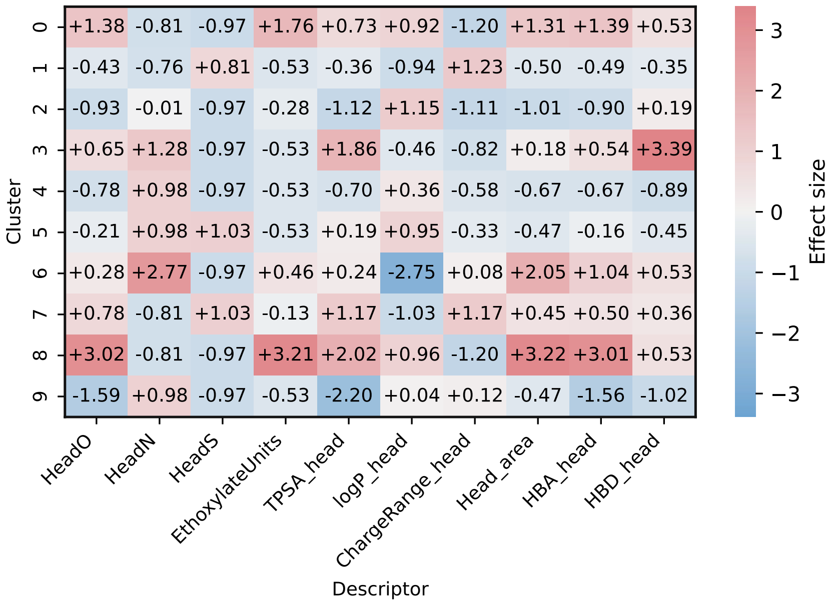

Figure S4. Descriptor effect-size profiles for head clusters in pC20 analysis. Heatmap showing the standardized effect size of each descriptor across clusters. Positive values (red) indicate that a descriptor is overrepresented within a given cluster relative to the full dataset, whereas negative values (blue) indicate underrepresentation. The magnitude of the effect size reflects the discriminative strength of each descriptor, highlighting the key physicochemical features that define and differentiate the structural characteristics of each cluster.

Chemical interpretation of head clusters (H0–H9)

- H0: nonionic ethoxylated oxygen-rich heads (high EO/HBA/HeadO; polyethoxy-like).
- H1: highly ionic sulfur-containing heads (high charge-range, sulfate-like; low head logP).
- H2: small low-polarity nonionic heads (high head logP, low TPSA/charge-range).
- H3: strongly hydrophilic, H-bond-donor-rich heads (high HBD/TPSA; amide/sugar-like).
- H4: N-containing compact ionic/zwitterionic heads (betaine-like signature; low HBD).
- H5: mixed N+S amphoteric/ionic heads with relatively higher hydrophobic character (higher head logP).
- H6: large bis-cationic/polyether-like heads (very high HeadN and head area, low head logP).
- H7: sulfur-rich anionic ethoxylated heads (high charge-range/TPSA with S-containing chemistry).
- H8: ultra-ethoxylated very large oxygenated heads (extreme EO, HeadO, HBA, and head area).
- H9: aromatic cationic low-polarity heads (very low TPSA/HeadO/HBA; pyridinium-like motif).

Table S5: Representative Structures of Tail Clusters in pC20 Analysis

Cluster ID	Representative Tail
0	CCCCCCCCCCCC
1	CCCCCCCCCCCCc1ccccc1
2	CCCCCCCCCCCCCCC
3	FC(F)C(F)(F)C(F)(F)C(F)(F)C(F)(F)C(F)(F)C(F)(F)C(F)(F)F
4	CCCCCCCCCCC.CCCCCCCCCCCC
5	CCCCCCCCC

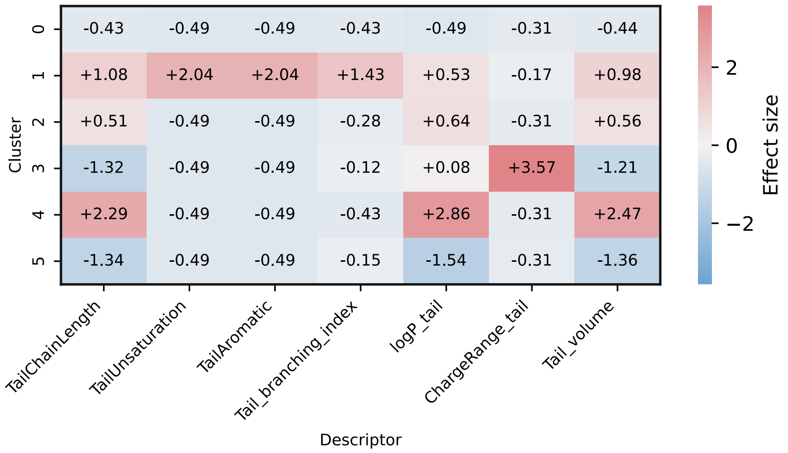

Figure S5. Descriptor effect-size profiles for tail clusters in pC20 analysis. Heatmap showing the standardized effect size of each descriptor across clusters. Positive values (red) indicate that a descriptor is overrepresented within a given cluster relative to the full dataset, whereas negative values (blue) indicate underrepresentation. The magnitude of the effect size reflects the discriminative strength of each descriptor, highlighting the key physicochemical features that define and differentiate the structural characteristics of each cluster.

Chemical interpretation of tail clusters (T0–T5, pC20 dataset)

- T0: linear saturated non-aromatic tails with relatively low hydrophobicity (lower logP/volume/length).
- T1: aromatic/unsaturated/branched tails (rigid hydrophobes with high structural complexity).
- T2: long linear alkyl tails with moderately high hydrophobicity (higher logP/volume/length).
- T3: highly polar/charged tails with short effective chain metrics (high ChargeRange_tail; fluorinated/charged-like signature).
- T4: very hydrophobic bulky long tails (highest logP, volume, and chain-length features).
- T5: short/light tails with low hydrophobicity (lowest logP/volume/length; fragmented/short-chain profile).

Surface Tension Analysis

Table S6: Representative Structures of Head Clusters in Surface Tension Analysis

Cluster ID	Representative Head
**0**	CNC(=O)[C@H](O)[C@@H](O)[C@H](O)[C@H](O)CO
**1**	OCCOCCO
**2**	O=S(=O)([O-])OCCO
**3**	OCCOCCOCCOCCOCCOCCOCCOCCOCCOCCOCCOCCOCCOCCOCCO
**4**	CNC(=O)[C@H](O)[C@@H](O)[C@H](OC1OC(O)C(O)C(O)C1O)[C@H](O)CO
**5**	C[N+](C)CC(=O)[O-]
**6**	C[n+]1ccccc1
**7**	OCCOCCOCCOCCOCCOCCOCCOCCO

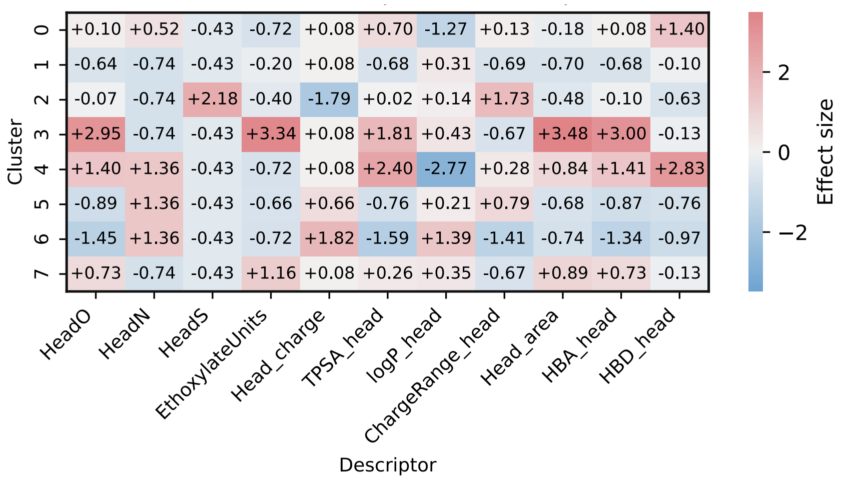

Figure S6. Descriptor effect-size profiles for head clusters in surface tension analysis. Heatmap showing the standardized effect size of each descriptor across clusters. Positive values (red) indicate that a descriptor is overrepresented within a given cluster relative to the full dataset, whereas negative values (blue) indicate underrepresentation. The magnitude of the effect size reflects the discriminative strength of each descriptor, highlighting the key physicochemical features that define and differentiate the structural characteristics of each cluster.

Chemical interpretation of head clusters (H0–H7)

- H0: highly polar, hydrogen-bond-rich heads (high HBD, low logP), consistent with sugar/amide-like chemistry.
- H1: compact, low-charge-range, N-poor polyether-like heads (small nonionic ethoxy-type motifs).
- H2: sulfur-containing anionic heads with strong charge-separation character (sulfate/sulfonate-like signature).
- H3: large, highly ethoxylated nonionic heads (high EO units, high head area, high HBA; strongly hydrated).
- H4: very hydrophilic amide/carbohydrate-like heads (high HBD and TPSA, low logP).
- H5: N-rich ionic/zwitterionic-like heads (quaternary ammonium–carboxylate/betaine-type pattern).
- H6: charged aromatic cationic heads with relatively lower oxygenation/polar surface (heteroaromatic cation-like).
- H7: medium-to-high EO nonionic heads with enlarged hydrated headgroup area.

Table S7: Representative Structures of Tail Clusters in Surface Tension Analysis

Cluster ID	Representative Tail
**0**	CCCCCCCCCCCC
**1**	CCCCCC(C)(C)c1ccccc1
**2**	CCCCCCCC
**3**	CCCCCCCCCCCCCCCC
**4**	CCCCCCCCCC
**5**	c1ccc(OCCCCCCOc2ccccc2)cc1

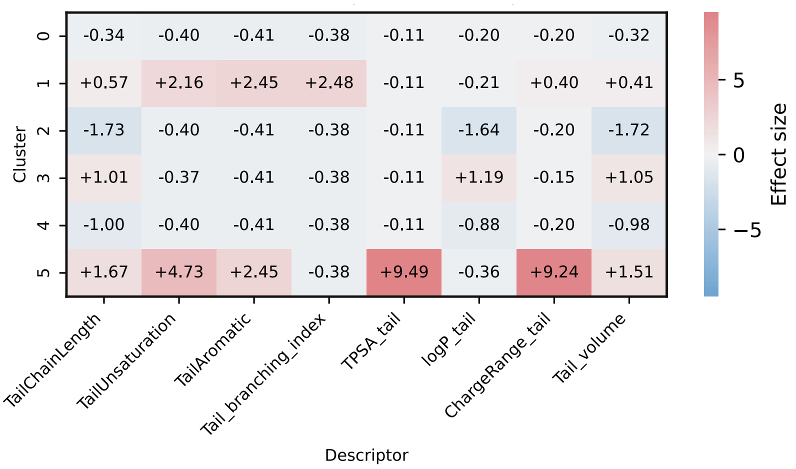

Figure S7. Descriptor effect-size profiles for tail clusters in surface tension analysis. Heatmap showing the standardized effect size of each descriptor across clusters. Positive values (red) indicate that a descriptor is overrepresented within a given cluster relative to the full dataset, whereas negative values (blue) indicate underrepresentation. The magnitude of the effect size reflects the discriminative strength of each descriptor, highlighting the key physicochemical features that define and differentiate the structural characteristics of each cluster.

Chemical interpretation of tail clusters (T0–T5)

- T0: linear, saturated, non-aromatic aliphatic tails (medium-length hydrocarbon character).
- T1: branched/aromatic/unsaturated tails (structurally rigid, bulky hydrophobes with aromatic contribution).
- T2: short linear saturated tails (low chain length/volume; weaker hydrophobic tail domain).
- T3: long linear saturated tails with high logP and volume (strong hydrophobic/lipophilic character).
- T4: short-to-intermediate linear saturated tails (similar class to T2, but slightly heavier aliphatic profile).
- T5: oxygenated aromatic tails with elevated tail polarity/charge-range (polar-aromatic tail chemistry distinct from pure alkyl tails).

OUTLIER DETECTION AND METACLUSTER REASSIGNMENT PROCEDURE

Each molecule is initially assigned to a metacluster h_i–t_j, defined by the combination of its head cluster $i$and tail cluster j. Within each metacluster, the within-group z-score of the target property is computed as:

$$z = \frac{x_a - \mu_{\mathrm{mc}}}{\sigma_{\mathrm{mc}}}$$

where $\mu_{\mathrm{mc}}$and σ_{mc} denote the mean and standard deviation of the target property within the corresponding metacluster. Molecules with $|z| \geq 2.5$ are flagged as potential outliers. While a threshold of 3 is commonly used for z-score-based outlier detection, a more conservative cutoff of 2.5 is adopted here to identify molecules whose target property deviates significantly from the metacluster distribution, while minimizing spurious detections, particularly in small groups[2].

For each detected outlier, a joint feature vector $X_{joint} = [X_{head} \parallel X_{tail}]$ is constructed by concatenating the preprocessed descriptor vectors of the head and tail groups. Euclidean distances between this vector and all metacluster centroids are then computed in the joint feature space, and candidate metaclusters are ranked in ascending order of distance.
For each candidate metacluster c(starting from the nearest and excluding the original assignment), a new z-score is evaluated as:

$$z_{new} = \frac{|x_a - \mu_c|}{\sigma_c}$$

The molecule is reassigned only if $z_{new} < z_{current}$, i.e., if the reassignment simultaneously improves structural proximity and reduces the statistical deviation of the target property. This dual criterion ensures that reassignment is physically meaningful: a molecule is relocated only if it is structurally closer to another metacluster and its property value is more consistent with that group.
If at least one reassignment occurs, the number of head or tail clusters (selected based on underrepresentation) is increased by one unit, and the procedure is repeated iteratively. A flowchart of the process is depicted in Figure S8.

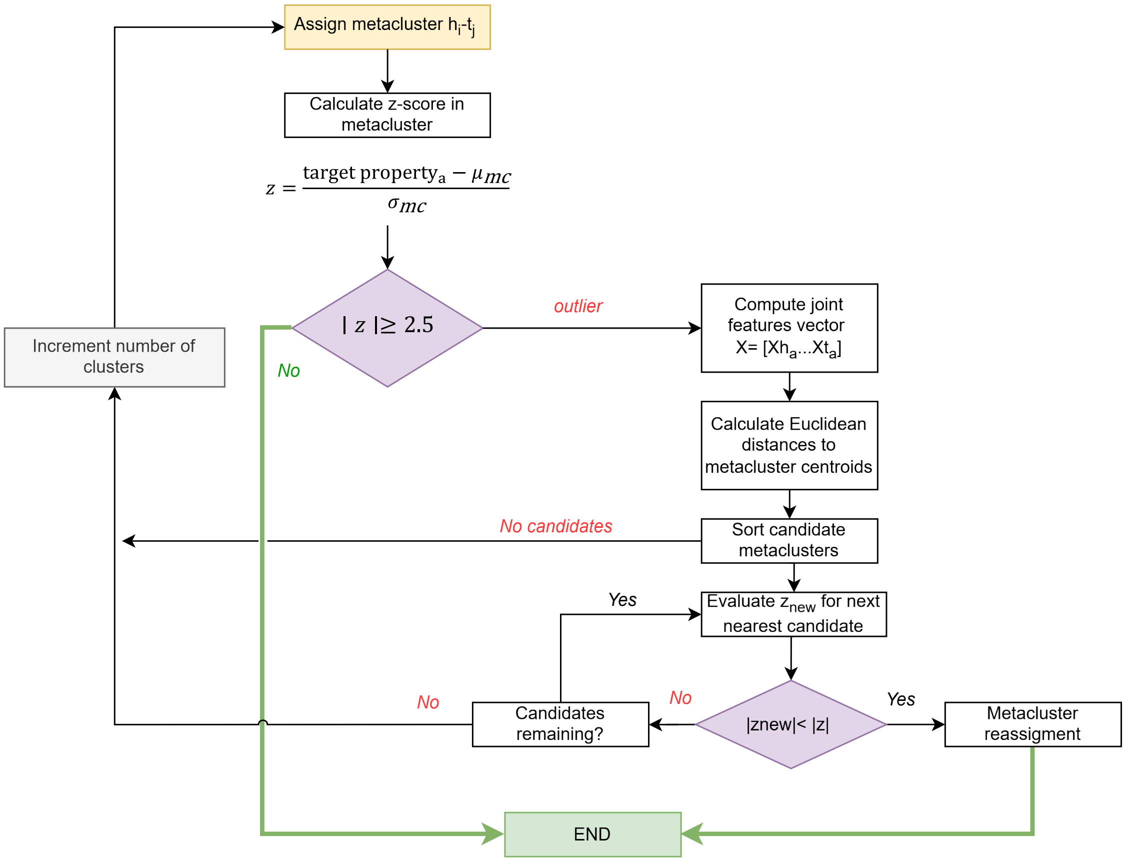

Figure S8. Flowchart of the outlier detection and metacluster reassignment procedure based on z-score and centroid distance. Each molecule a is assigned to a metacluster (mc) and its z-score is computed, where μ_{mc} and σ_{mc} are the mean and standard deviation of the target property within the metacluster.

REFERENCES

(1) Fizer, M.; Fizer, O. Theoretical Study on Charge Distribution in Cetylpyridinium Cationic Surfactant. *J. Mol. Model.* **2021**, *27* (7), 203.

(2) Chikodili, N. B.; Abdulmalik, M. D.; Abisoye, O. A.; Bashir, S. A. Outlier Detection in Multivariate Time Series Data Using a Fusion of K-Medoid, Standardized Euclidean Distance and Z-Score. In *International conference on information and communication technology and applications*; Springer, 2020; pp 259–271.
